# Supplementary material for: Drug-related problems among type 2 diabetic patients in Sunwal Municipality of Western Nepal
Source: PLoS One. 2024 Nov 21;19(11):e0309781. doi: 10.1371/journal.pone.0309781 (PMC11581294; doi:10.1371/journal.pone.0309781)
Supplement: Attachment — Submitted filename: Comments.docx [file pone.0309781.s002.docx]

1. The World Health Organization (WHO) defines Non-communicable diseases (NCDs) as chronic diseases which occur for a longer duration of time as a consequence of genetic, physiological, environmental, and behavioral factors and is responsible for about 77% of death in low and middle income countries (LMIC). It appears as if the definition of NCD requires the death figures. To avoid misinterpretation. Split the statement.
2. Replace Familial history by family history and type 2 diabetic mellitus by type 2 diabetes mellitusthroughout.
3. ‘Pretesting was done 95 at 10% of the study population and reliability was calculated using Cronbach Alpha. The Cronbach alpha for patient profile, diet, and physical activity was found to be 0.701 while it was 0.706 for DRPs’: The statement is not clear and needs to be evaluated by and expert statistician. Cronbach’s alpha is a measure of internal consistency in a set of items used for a questionnaire. Authors should mention how and on which specific items Cronbach alpha was calculated. If not sure, the statement should be deleted.
4. Lines 114-115: Glycaemic targets: Should be less than and not above the values given.
5. Table 3: Authors should correct the Anatomical class (WHO-ATC). For example: Alimentary canal should be replaced by Alimentary tract and metabolism. Likewise blood should be replaced by blood and blood forming organs.
6. In coloumn 3 of Table 3, Authors have randomly used Therapeutic class for some drugs and Chemical subgroup. Please keep uniformity and use either of them and not both. Common individual drugs could have been mentioned example metformin, telmisartan etc.
7. Table 8: Authors can only mention the PCNE Class to which DRPs were observed rather than mentioning each cause
8. Table 9: Authors should revise the table. Give the total number in each group and the number (%) developing DRPs. For example 86 of 55 years and above among whom 41(47.7%) developed DRPs. This will ease the interpretation of data for the readers.
9. Discussion: Needs a thorough revision. Not comprehensible in the present form.
10. Lines 199-202:Mention rates of DRPs in the studies cited (28 and 29 references) in %
11. Lines 205-207: 58.2% is not much higher than 53.3% rates of DRPs observed in the present study. Correct the statement. Yes it is higher than 88% rates of DRPs mentioned in the next statement by authors.
12. What were the major DRPs in < 60 years (adults) and ≥ 60 years of age.
13. Since a considerable percentage of participants were older adults (50 years of age and above ), Authors should discuss other studies on Drug related problems in older adults.
14. Contrary to the findings of the present study, one large study (mentioned below )has shown hypoglycemia due to antiDM drugs as the commonest metabolic abnormality also the second common DRP causing hospitalization in older patients.

Kaur U, Chakrabarti SS, Gupta GK, Singh A, Gambhir IS. Drug-related problems in older adults in outpatient settings: Results from a 6-year long prospective study in a tertiary hospital of north India. Geriatr Gerontol Int. 2023 Aug 14. doi: 10.1111/ggi.14650. Epub ahead of print. PMID: 37577765.

15. Lines 242-243: In our study the most common comorbid condition associated with T2DM was hypertension. Similar result was obtained in the study conducted by Huri & Ling, 2013[29] and Ogbonna et al, 2014: Should be deleted. Discussion should focus on relevant findings of the study and not on co-morbidities or baseline characteristics unless they are determinants of DRPs
16. Lines 249- 251: “. In this study oral hypoglycemic agents (OHA) and insulin contain (n=275; 55.5%) of prescription as 70.6 as monotherapy, (24.1%) combination and insulin (5.4%)”: Not clear. Please Clarify
17. Line: 244: There is a statistical association between the duration of diabetes and

the DRPs (*p=.007*). What does this signify? DRPs common in recently diagnosed or in patients with chronic diabetes.

18. One important variable which authors missed was number of drugs taken concomitantly.

Polypharmacy shares a direct relation with DRPs such as adverse drug reactions.

19. Likewise financial status and psychological factors, history of side effects are important factors deciding compliance to therapy and were missed.
20. Lines 263-264: Patients eating less fruits were found to develop DRPs commonly. Authrs should elaborate the association found between duration of diabetes and fruits consumption and its significance
21. Lines 273: Interventions were not planned and carried out while the patient counseling and referral to medical doctor was done.: Statement is not clear. Were any interventions planned at pharmacist level after diagnosing DRPs?
22. Higher female enrolment does not mean higher % of females developing DRPs. Lines 230-235 should be modified.
23. Write a separate Limitation section before Conclusion
24. Modify the Conclusion part accordingly. Avoid statistical term, unclear problems etc. in conclusion and give only clinically relevant findings and factors with important logical associations with DRPs
25. Authors should discuss only important findings in the Discussion and not each finding of Results. For ex : Lines 239-243, 244-253 seem redundant
26. That non compliance was the commonest cause of DRP should be followed by a statement on the need of proper intervention at the prescription stage or by a medical social worker for importance of treatment adherence. Important reasons for non-compliance should also be sought in future.
27. Any reason why other more common DRPs such as hypoglycaemia, hypotension and hypertension were not observed
28. Tables 2 and 4 can be deleted as data is there in text.
