# Supplementary material for: Drug-related problems among type 2 diabetic patients in Sunwal Municipality of Western Nepal
Source: PLoS One. 2024 Nov 21;19(11):e0309781. doi: 10.1371/journal.pone.0309781 (PMC11581294; doi:10.1371/journal.pone.0309781)
Supplement: Attachment — Submitted filename: Plos one DRPs.docx [file pone.0309781.s004.docx]

Authors have made significant modifications. There are some issues in the revised manuscript that need clarification from the authors.

Please find them below:

1.Anatomical system is still not defined appropriately. Authors are advised to go through the WHO-ATC classification and quote the system as mentioned in the WHO-ATC system. For example: It should be Cardiovascular system and genitourinary system and sex hormones. The alimentary canal is still mentioned at certain points in the text.

2.‘Major Therapeutic Class of Drugs Used by Patients’: Authors have not provided the denominator (total number of drugs) from which percentages are calculated.

3.Duration of diabetes is also inadequately described. The sum of individuals is not matching with 182.

4. PCNE C7.1‘Patient doesnot take drug at all and C8 Non-compliance, I understand , they are interrelated. But to bring clarity to readers, authors should mention to which group they categorized the Patients not adhering to drug therapy. Was it considered under C7.1 or C8

5.The present study did not find any association of DRPS with gender. Hence authors should remove the statement ‘ Among the 97 patients with DRPs, the occurrence of DRP was more common among females’ written in the Discussion section.

6.The discussion part on fruit consumption is wrongly interpreted by the authors. DRPs were more common in the group eating less servings of fruits.

7.A significant % of included individuals were older adults (50-60 years). Authors have still not mentioned the fact that they didn't observe adverse drug reactions as the common DRPs. ADRs are the commonest DRPs. Though authors mention in lines 274-276 that they did not observe any association of DRPs with age, they should specifically mention that ADRs were not a common observation and lend a suitable explanation for the same.

8.In Abstract: Authors mention the biguanides and sulfonylureas to be responsible for the DRPs. Just because these were the common classes prescribed doesnot mean they are associated with DRPs as head to head comparisons between the rates of DRPs with the two classes are not described by the paper. Rather, authors should mention biguanides and sulfonylureas were the common classes of drugs prescribed.

9.Likewise, authors should state **Drug classes associated with DRPs** and **not** causing DRPs in Tables such as Table 4

10.‘Diabetic’ word can be removed from the sentences where ‘patients with type 2 diabetes’ are mentioned

11.The entire text needs Grammar correction.
