# Supplementary material for: Drug-related problems among type 2 diabetic patients in Sunwal Municipality of Western Nepal
Source: PLoS One. 2024 Nov 21;19(11):e0309781. doi: 10.1371/journal.pone.0309781 (PMC11581294; doi:10.1371/journal.pone.0309781)
Supplement: Attachment — Submitted filename: Response to Reviewers.docx [file pone.0309781.s005.docx]

Review Comments to the Author

Reviewer #2: Authors have made significant modifications. There are some issues in the revised manuscript that need clarification from the authors.

Please find them below:

1. Anatomical system is still not defined appropriately. Authors are advised to go through the WHO-ATC classification and quote the system as mentioned in the WHO-ATC system. For example: It should be Cardiovascular system and genitourinary system and sex hormones. The alimentary canal is still mentioned at certain points in the text.

**Response to reviewer:** We have now defined the term and modified the text and table as suggested. We have used the reference (<https://atcddd.fhi.no/atc_ddd_index/>) to make the ATC classification for the therapeutic classification of drugs used by patients which totally aligns with the reviewer’s comments too. [Addressed in Line numbers 178-182]

2.‘Major Therapeutic Class of Drugs Used by Patients’: Authors have not provided the denominator (total number of drugs) from which percentages are calculated.

**Response to reviewer:** We have now mentioned the total number of drugs (i.e. n=679) from which the percentages are calculated. [Addressed as in Table 2]

3. Duration of diabetes is also inadequately described. The sum of individuals is not matching with 182.

**Response to reviewer:** We have now added the meaning for the duration of diabetes. We meant it for how long the patient had diabetes. It was divided into recently diagnosed, (who were recently diagnosed as per laboratory confirmation) and patients with diabetes for less than 2 years, 2-5 years, 5-10 years and more than 10 years. The sum is 182. [Addressed in Line numbers 229-231]

4. PCNE C7.1‘Patient does not take drug at all and C8 Non-compliance, I understand, they are interrelated. But to bring clarity to readers, authors should mention to which group they categorized the Patients not adhering to drug therapy. Was it considered under C7.1 or C8

**Response to reviewer:** Thank you for your comment. We categorized patients who are not taking their medication without any apparent reason under category C7.1. Those who are not taking their medication due to specific reasons, such as the cost of treatment, lack of information about administration, forgetting to take the medication, demographic factors (e.g., unavailability of medical services in rural areas), side effects of drugs, etc., were categorized under C8.

After considering your comments, we discussed this categorization in detail and concluded that regardless of the reasons or conditions, not taking or taking less medication is related to patient behaviour and should be classified under C7. Since both categories (C7.1 and C8) represent aspects of non-compliance, and to avoid confusion for readers, we decided to merge these two categories. We have now combined the values for C7.1 and C8 under a single category, C7.1, and have updated the relevant sections in the table, results, discussion, and metadata accordingly.

5. The present study did not find any association of DRPs with gender. Hence authors should remove the statement ‘Among the 97 patients with DRPs, the occurrence of DRP was more common among females’ written in the Discussion section.

**Response to reviewer:** Thank you for the suggestion. We have now removed this sentence from the discussions section. [Addressed in Line numbers 288-289].

6. The discussion part on fruit consumption is wrongly interpreted by the authors. DRPs were more common in the group eating less servings of fruits.

**Response to reviewer:** Thank you for the comment, We have now corrected this interpretation. [Addressed in line number 326-329.

7. A significant % of included individuals were older adults (50-60 years). Authors have still not mentioned the fact that they didn't observe adverse drug reactions as the common DRPs. ADRs are the commonest DRPs. Though authors mention in lines 274-276 that they did not observe any association of DRPs with age, they should specifically mention that ADRs were not a common observation and lend a suitable explanation for the same.

**Response to reviewer:** Thank you for underlining this deficiency. We have now addressed this issue. [Addressed in Line numbers 293-298].

8. In Abstract: Authors mention the biguanides and sulfonylureas to be responsible for the DRPs. Just because these were the common classes prescribed does not mean they are associated with DRPs as head-to-head comparisons between the rates of DRPs with the two classes are not described by the paper. Rather, authors should mention biguanides and sulfonylureas were the common classes of drugs prescribed.

**Response to reviewer:** Thank you for highlighting this deficiency and suggestion. We have now added the sentence in the abstract section. [Addressed in Line number 43]

9. Likewise, authors should state Drug classes associated with DRPs and not causing DRPs in Tables such as Table 4

**Response to reviewer:** Thank you for highlighting this and your suggestion. We have now changed the “Drug classes associated with DRPs and not causing DRPs. We have replaced the word ‘drug causing’ with drugs associated too. [Addressed in Table 4]

10. ‘Diabetic’ word can be removed from the sentences where ‘patients with type 2 diabetes’ are mentioned

**Response to reviewer:** We have now removed the word diabetic from the sentence where ‘patient with type 2 diabetes’ is mentioned. [Line 36]

11. The entire text needs Grammar correction

**Response to reviewer:** Thank you for your feedback regarding the grammar in the manuscript. We have now utilized the services of a professional English language editing service to correct the grammatical errors throughout the manuscript. They have also assured us that they are ready to re-edit the text if any further language changes are required to meet the journal's standards.

We have restructured some of the sentences, Table 1 and Table 3.
